# EVpedia: an integrated database of high-throughput data for systemic analyses of extracellular vesicles

**DOI:** 10.3402/jev.v2i0.20384

**Published:** 2013-03-19

**Authors:** Dae-Kyum Kim, Byeongsoo Kang, Oh Youn Kim, Dong-sic Choi, Jaewook Lee, Sae Rom Kim, Gyeongyun Go, Yae Jin Yoon, Ji Hyun Kim, Su Chul Jang, Kyong-Su Park, Eun-Jeong Choi, Kwang Pyo Kim, Dominic M. Desiderio, Yoon-Keun Kim, Jan Lötvall, Daehee Hwang, Yong Song Gho

**Affiliations:** 1 Department of Life Science, Division of Molecular and Life Sciences, Pohang University of Science and Technology, Pohang, Republic of Korea; 2 School of Interdisciplinary Bioscience and Bioengineering, Pohang University of Science and Technology, Pohang, Republic of Korea; 3 Department of Molecular Biotechnology, WCU Program, Konkuk University, Seoul, Republic of Korea; 4 Department of Neurology, The University of Tennessee Health Science Center, Memphis, USA; 5 Department of Molecular Science, The University of Tennessee Health Science Center, Memphis, USA; 6 The Charles B. Stout Neuroscience Mass Spectrometry Laboratory, The University of Tennessee Health Science Center, Memphis, USA; 7 Department of Internal Medicine, Krefting Research Centre, University of Gothenburg, Gothenburg, Sweden

**Keywords:** nanocosmos, communicasomes, exosomes, microvesicles, outer membrane vesicles, membrane vesicles, web portals, phylogenetic analyses

## Abstract

Secretion of extracellular vesicles is a general cellular activity that spans the range from simple unicellular organisms (e.g. archaea; Gram-positive and Gram-negative bacteria) to complex multicellular ones, suggesting that this extracellular vesicle-mediated communication is evolutionarily conserved. Extracellular vesicles are spherical bilayered proteolipids with a mean diameter of 20–1,000 nm, which are known to contain various bioactive molecules including proteins, lipids, and nucleic acids. Here, we present EVpedia, which is an integrated database of high-throughput datasets from prokaryotic and eukaryotic extracellular vesicles. EVpedia provides high-throughput datasets of vesicular components (proteins, mRNAs, miRNAs, and lipids) present on prokaryotic, non-mammalian eukaryotic, and mammalian extracellular vesicles. In addition, EVpedia also provides an array of tools, such as the search and browse of vesicular components, Gene Ontology enrichment analysis, network analysis of vesicular proteins and mRNAs, and a comparison of vesicular datasets by ortholog identification. Moreover, publications on extracellular vesicle studies are listed in the database. This free web-based database of EVpedia (http://evpedia.info) might serve as a fundamental repository to stimulate the advancement of extracellular vesicle studies and to elucidate the novel functions of these complex extracellular organelles.

Communication between cells and the environment is essential for single and multicellular organisms. Almost all kinds of simple unicellular organisms (e.g. archaea; Gram-positive and Gram-negative bacteria) to complex multicellular ones secrete nano-sized extracellular vesicles (EVs) for their intercellular communications (1–4). These membrane vesicles are spherical bilayered proteolipids with a mean diameter of 20–1,000 nm (2, 3, 5, 6), which are known to contain various bioactive molecules, including proteins, nucleic acids (mRNA, microRNA, rRNA, and tRNA), and lipids (3, 6–11). EVs derived from archaea and Gram-positive bacteria are called membrane vesicles (1, 2), whereas Gram-negative bacterial EVs are called outer membrane vesicles (3). Mammalian cells secrete exosomes and ectosomes (also known as microvesicles) either constitutively or in a regulated manner (4).

Although EVs play several physiological and pathological functions (12, 13), and occupy an emerging position in the field of biomarker discovery (14, 15), it is difficult to study EVs because they contain various bioactive molecules. Recently, however, to solve this problem, high-throughput analyses were performed on prokaryotic and eukaryotic EVs. For example, many mass spectrometry-based proteomic studies, microarray or next-generation sequencing-based transcriptomic studies, and chromatography-based lipidomic studies on EVs are reported (16–18). Over 200,000 vesicular proteins, mRNAs, miRNAs, and lipids identified in non-mammalian eukaryotic and mammalian EVs, have been compiled in the Exocarta database (16, 17), which was recently updated into Vesiclepedia (http://www.microvesicles.org; 18). However, there has been no resource on vesicular components (proteins, nucleic acids, and lipids) derived from diverse types of prokaryotic and eukaryotic cells. In addition, an analytical tool for their Gene Ontology enrichment analyses, network analyses of vesicular proteins and mRNAs, and a comparison of vesicular datasets by ortholog identification have not been developed. These types of systematic analyses on vesicular components provide global insights into the mechanisms involved in vesicular cargo-sorting and EV biogenesis as well as the pathophysiological roles of EVs. For example, we recently showed that mammalian vesicular proteins are physically and functionally interconnected to form functional modules involved in EV biogenesis and functions; those data suggest that an EV is a nano-sized extracellular organelle (i.e. nanocosmos) rather than a cellular dust (19).

Here, we present EVpedia (http://evpedia.info), which is an integrated database of high-throughput datasets from EVs launched in January of 2012 with the latest update in November of 2012 (Fig. 1). EVpedia provides information on proteins, mRNAs, miRNAs, and lipids enclosed in prokaryotic, non-mammalian eukaryotic, and mammalian EVs. Moreover, EVpedia also provides an array of tools, such as the search and browse of vesicular components, Gene Ontology enrichment analysis, network analysis of vesicular components, and a comparison of vesicular datasets by ortholog identification. In addition, publications on EV studies are listed in the database. This free web-based database of EVpedia might serve as a fundamental repository to stimulate the advancement of EV studies and to elucidate the novel functions of these complex extracellular organelles.

**Fig. 1:**
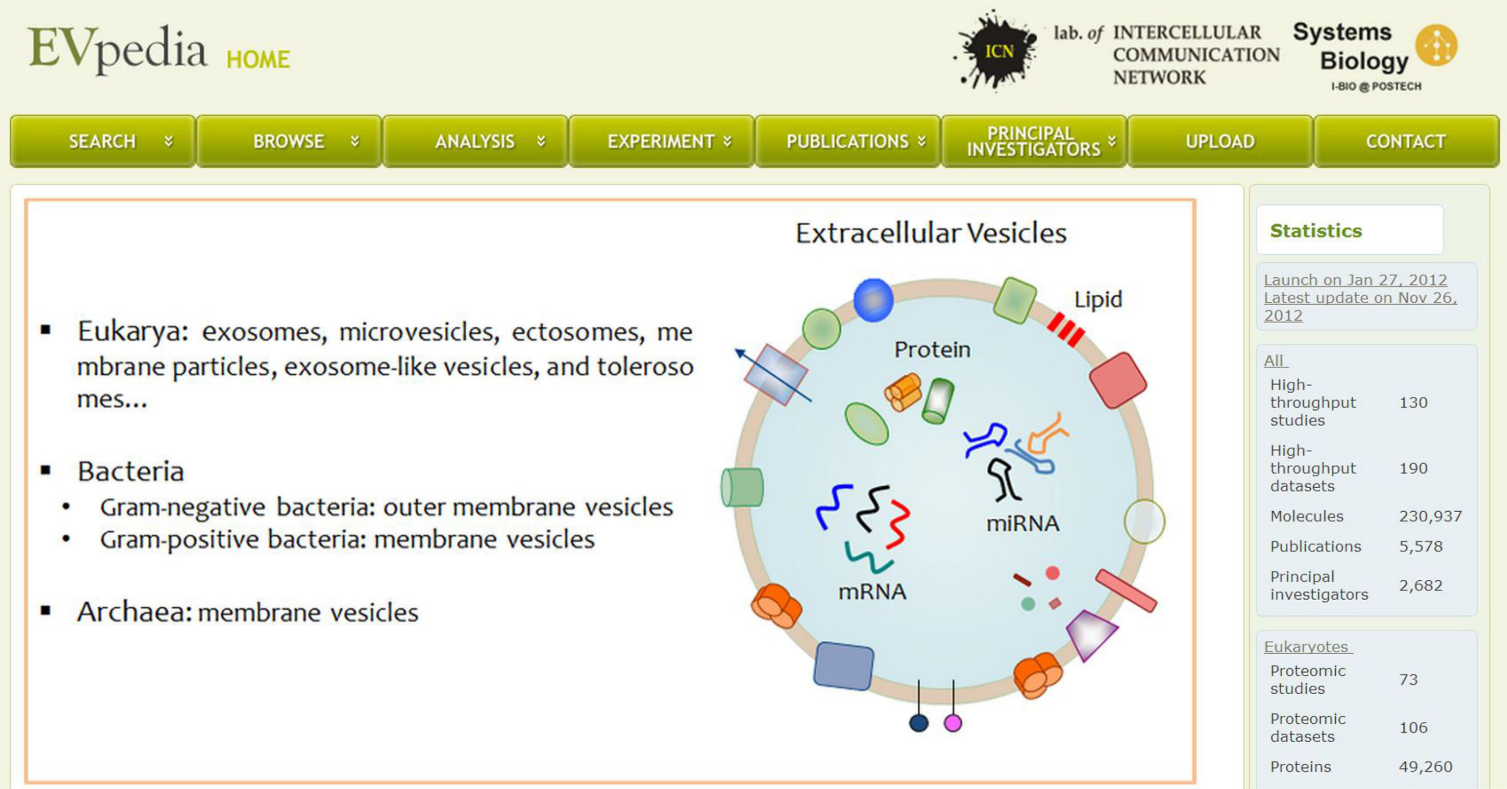
Snapshot of EVpedia homepage (http://evpedia.info). General introduction of EVs and statistics of EVpedia are provided in the “Home” menu.

## Overall structure of EVpedia

For a systematic exploration of high-throughput datasets from prokaryotic and eukaryotic EVs, EVpedia has four functional modules: (a) database of high-throughput datasets, (b) search and browse of database, (c) identification of orthologous vesicular proteins and (d) bioinformatic analyses of vesicular components.

A total of 230,937 vesicular components from 190 high-throughput datasets from 130 high-throughput studies were collected in the current EVpedia database (Table I). Among the 190 high-throughput datasets, 166 derived from eukaryotes and 24 from prokaryotes. Vesicular high-throughput datasets, detailed methods for EV isolation, and high-throughput analysis for each dataset were arranged as tables under the “Experiment” menu. User-requested lists of vesicular components are provided in the search and browse function. A new vesicular high-throughput dataset can be submitted to the database by the “Upload” menu.

**Table I:** Statistics of EVpedia, Exocarta, and Vesiclepedia

	EVpedia			Exocarta	Vesiclepedia
		
	All	Eukaryotes	Prokaryotes	Eukaryotes	Eukaryotes
Publications	5,578	4,847	731	–	–
High-throughput proteomes^a^					
Studies	90	73	17	15	25
Datasets	129	106	23	19	32
Proteins	52,786	49,260	3,526	9,554	16,789
High-throughput transcriptomes					
mRNA					
Studies	15	15	0	2	4
Datasets	19	19	0	3	5
mRNAs	164,519	164,519	0	2,307	19,353
miRNA					
Studies	9	9	0	4	9
Datasets	22	22	0	11	18
miRNAs	13,287	13,287	0	739	913
Lipidomes					
Studies	16	15	1	9	11
Datasets	20	19	1	10	13
Lipids	345	329	16	190	201

a High-throughput proteomes in which at least 50 vesicular proteins were identified (5).

To analyze protein or mRNA lists, EVpedia provides Gene Ontology enrichment analysis and network analysis. In addition, for comparison of vesicular proteome and transcriptome (mRNA) from different strains, we provide ortholog identification among those species in EVpedia. Based on the ortholog information, one can compare lists of vesicular proteins or mRNAs among different species with set analysis. For all the analyses, the molecule lists from the EVpedia and the new molecule lists from the users are both applicable.

An overall comparison of EVpedia with Exocarta and Vesiclepedia is shown in Tables I and II. These three web-based repositories contain proteomic, transcriptomic and lipidomic studies of non-mammalian eukaryotic and mammalian EVs. However, only EVpedia provides additional proteomic and lipidomic studies on prokaryotic EVs (Table I). Moreover, EVpedia also provides the information on EV-related publications (Table I) as well as an array of analytic tools (Table II), including (a) Gene Ontology enrichment analysis of vesicular components, (b) network analyses of vesicular components and (c) set analysis – a comparison of vesicular proteome and transcriptome data sets with ortholog identification. Currently, EVpedia collects vesicular proteomes identified only with high-throughput studies, but not immunoblotting or immunoelectron microscopy. We will expand the EVpedia database by adding these low-throughput protein datasets to address the hypothesis-driven or biological questions on EVs.

**Table II:** Analytic tools in EVpedia, Exocarta, and Vesiclepedia

	EVpedia	Exocarta	Vesiclepedia
Search			
Protein search	O	O	O
Sequence search	O	X	X
Browse	O	O	O
Analysis			
Gene Ontology enrichment analysis	O	X	X
Network analysis	O	X	X
Set analysis	O	X	X
Experiment			
Isolation strategy	O	O	O
High-throughput analysis strategy	O	O	O
PubMed link	O	O	O
Upload	O	X	X

## Search and browse of EVpedia

**Fig. 2:**
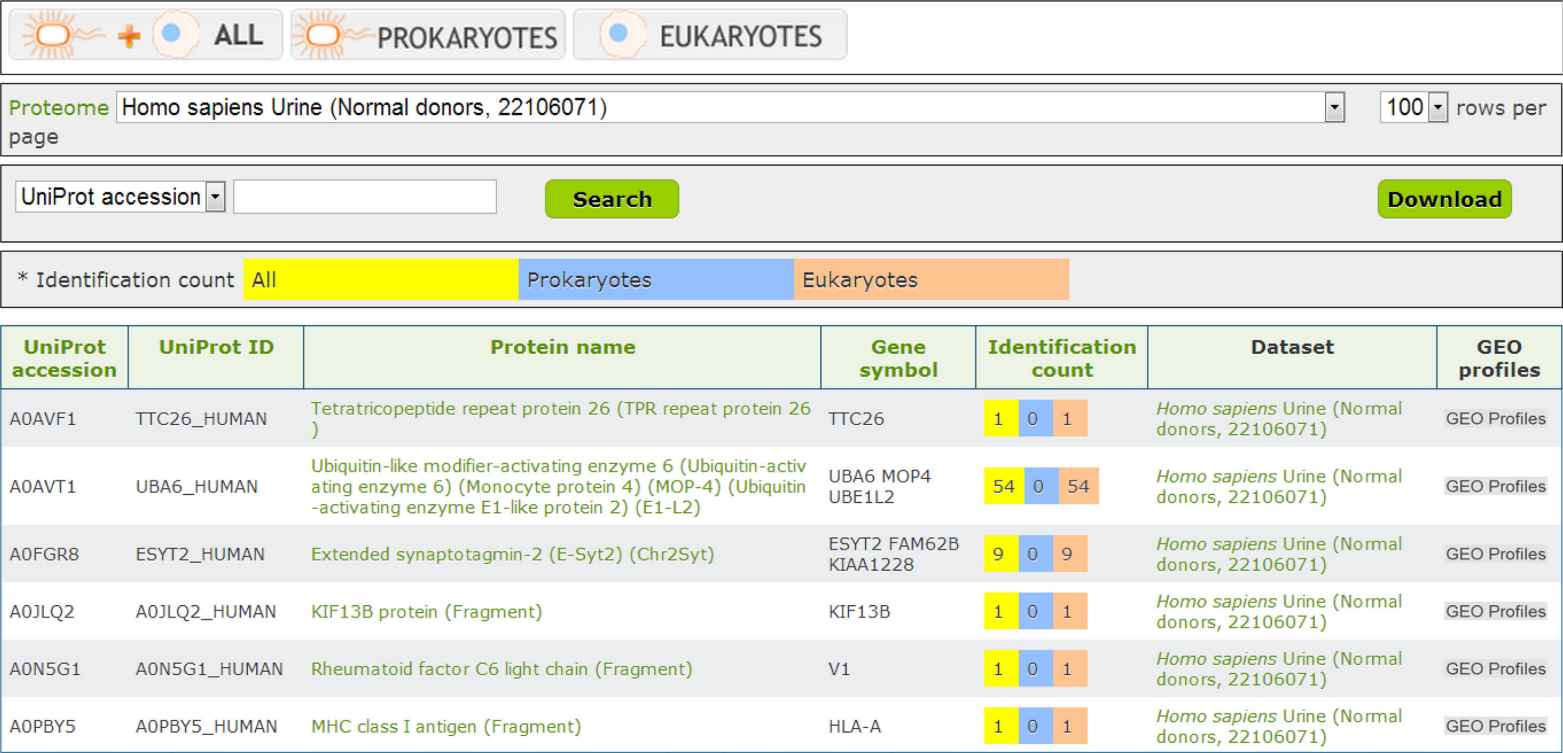
Browse function of EVpedia. With “Browse” menu, the protein list from the individual dataset or all the datasets can be browsed. In this figure, we browsed the vesicular proteins of the urine from normal donors (20).

In the “Search” menu, EVpedia provides two web interfaces: (a) search with protein names, UniProt accessions, or UniProt IDs (“Search - Protein search” menu) and (b) search with amino acid sequences in the plain format (“Search - Sequence search” menu). In addition, all vesicular components in EVpedia can be browsed in the “Browse” (Fig. 2) and the “Experiment” menu, and these tables can be downloaded in the tab-separated values (TSV) format.

## Gene ontology enrichment, network, and set analyses of EVpedia

The “Analysis” menu in EVpedia provides an array of bioinformatic analysis tools: (a) Gene Ontology enrichment and network analyses of the vesicular components and (b) set analysis among more than two different sets of the vesicular proteins or mRNAs through ortholog identification.

**Fig. 3:**
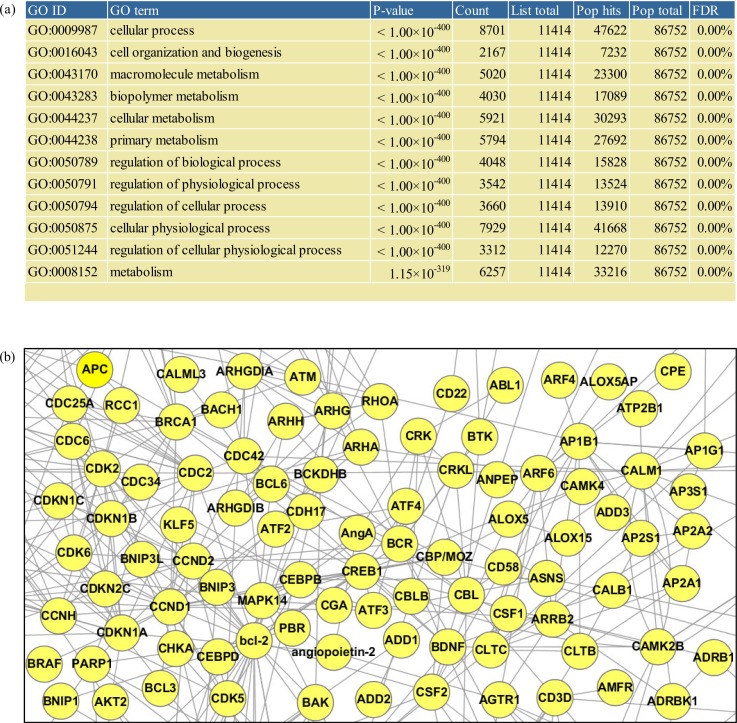
Gene Ontology enrichment and network analyses. All analyses in this figure involved mRNA transcriptome of EVs derived from *Homo sapiens* mast cell HMC-1 (21). In the “Analysis – Gene Ontology enrichment analysis” menu (a), by defining the species from which the analyzed list of proteins originates, the type of Gene Ontology terms (i.e. biological process, molecular function, and cellular component), and the cut-off of the enrichment p-value, the Gene Ontology enrichment analysis can be performed. In “Analysis – Network analysis” menu (b), by defining the species to which the functional interactome data belong, the number of additional nodes, and confidence of the interactome data, one can perform the network analysis of a protein list in EVpedia.

Through Gene Ontology enrichment analysis is in the “Analysis – Gene Ontology enrichment analysis” menu, the enriched terms (i.e. Gene Ontology biological process, molecular function, and cellular component) of vesicular components can be obtained (e.g. mRNAs in Fig. 3a). Via network analysis in the “Analysis – Network analysis” menu, functional relationships among vesicular components can be drawn into biological networks (e.g. mRNAs in Fig. 3b).

**Fig. 4:**
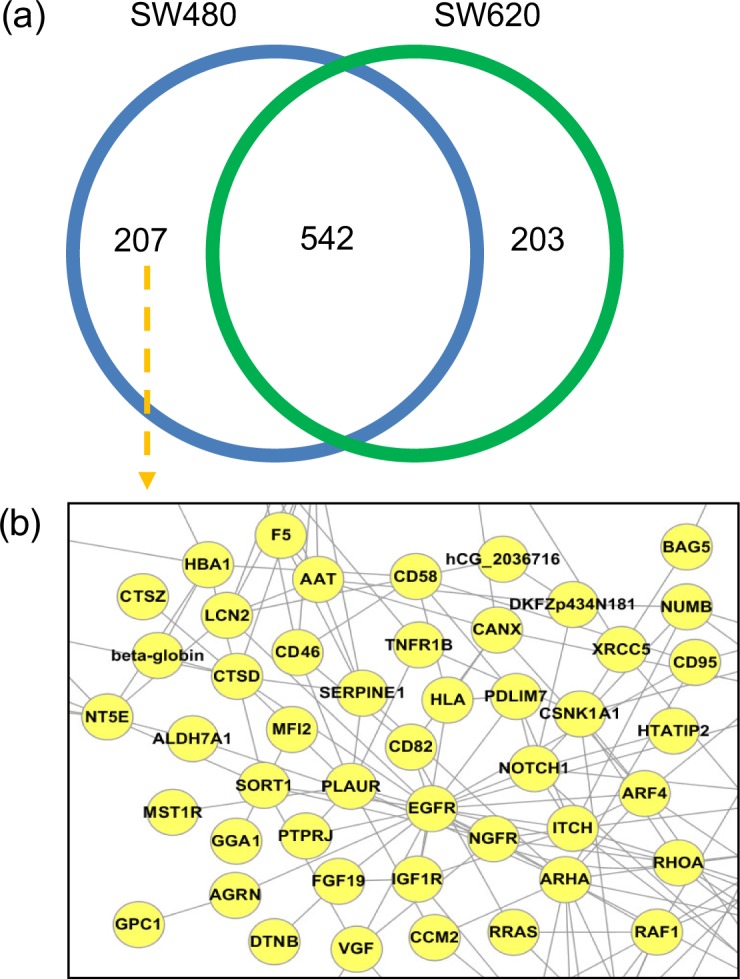
Set analysis of EVpedia. In “Analysis – Set analysis” menu, the dataset of the “proteome+transcriptome (mRNA)”, “transcriptome (miRNA)”, and “lipidome” can be chosen for set analysis. Especially for the datasets of “proteome+transcriptome (mRNA)”, information from ortholog identification is used to build the Venn diagram (a). For example, the Venn diagram is drawn by comparing the ortholog clusters of vesicular proteomes from *Homo sapiens* SW480 and SW620 colorectal cancer cells (20). The functional network of SW480-specific vesicular proteins can be drawn (b).

Moreover, EVpedia provides comparative analyses among more than two different sets of vesicular components by the “Analysis – Set analysis” menu. For example, we selected two sets of vesicular proteins (22): *Homo sapiens* colorectal cancer cell SW480 and SW620 (Fig. 4). The Venn diagram in Fig. 4a shows the number of members in the set intersection and the set difference between SW480 and SW620. The lists of each subset in the Venn diagram can be obtained for further analyses, such as the network analysis (Fig. 4b). Note that all of these analyses can be applied to a new list of proteins or mRNAs, including a newly uploaded vesicular proteome or mRNA transcriptome.

## EV-related publications in EVpedia

Publications on prokaryotic and eukaryotic EVs are manually curated and stored in the “Publications” menu. With NCBI PubMed search (http://www.ncbi.nlm.nih.gov/pubmed) for text-mining solution, we collected candidate papers related to prokaryotic and eukaryotic EVs: using argosome*, “blebbing vesicle”, “blebbing vesicles”, “budding vesicle”, “budding vesicles”, dexosome*, ectosome*, “extracellular vesicle”, “extracellular vesicles”, exosome*, exovesicle*, “matrix vesicle”, “matrix vesicles”, microparticle*, microvesicle*, “membrane particle”, “membrane particles”, “membrane vesicle”, “membrane vesicles”, nanovesicle*, oncosome*, “outer membrane bleb”, “outer membrane blebs”, prostasome*, “shedding vesicle”, “shedding vesicles”, and tolerosome* as search parameters. All searched publications were manually reviewed to verify whether they are genuinely related to EVs. For example, studies on exosomes of RNA degradation activity (23) were excluded. More detailed information, such as bibliographies, authors, and abstracts was excerpted from NCBI PubMed with automatized Python code (version 2.7.3).

**Fig. 5:**
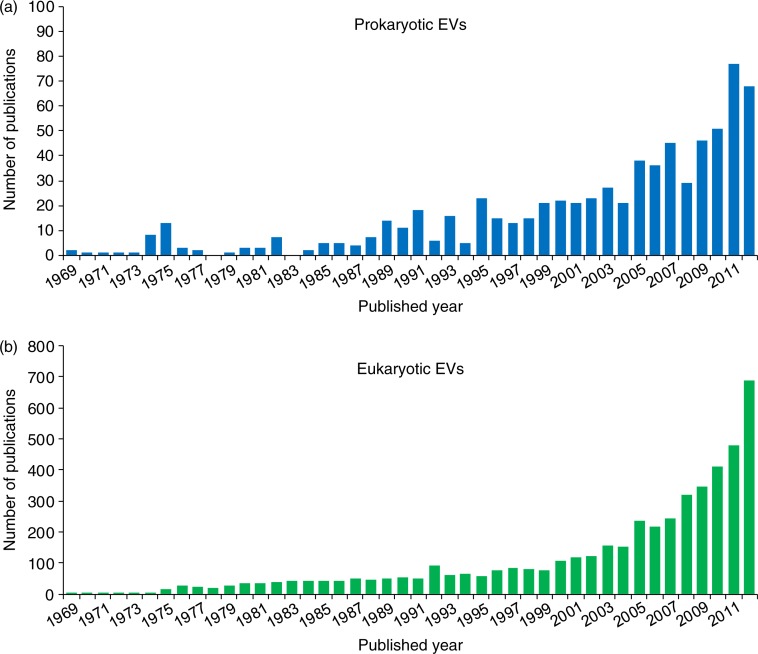
Publications in EVpedia. In “Publications” menu, one can browse papers related to prokaryotic (a) and eukaryotic (b) EVs. The bar graph shows the number of EV publications for each year. One can search the papers with a keyword in the type of “category”, “bibliography”, “title”, “author”, and “title+abstract”.

As shown in Fig. 5, the number of studies on prokaryotic and eukaryotic EVs is growing rapidly; this rapid growth indicates that the field of EVs is expanding intensively. In addition, the major principal investigators published a paper on EVs are listed with their EV-study publications as tables in the “Principal investigators” menu. The users can survey the major researchers and their research fields to provide more insights on their EV studies.

## System requirements of EVpedia

When building the EVpedia, we followed the international web standards that are compatible with most existing systems for web browsing. However, we recommend the following system requirements for best performance; operating system – MS Windows 7; internet browser – Google Chrome; resolution – 1,280×1,024.

We have tested the performance of EVpedia on the following systems; operating systems—MS Windows XP/7 and Apple OS X for personal computers, Google Android and Apple iOS for cell phones and tabloids; internet browsers – Google Chrome, Microsoft Internet Explorer, Apple Safari, and Mozilla Firefox. For network analysis, EVpedia requires Java Web Start (http://www.oracle.com/technetwork/java/javase/downloads/index.html).

## Conclusion and future directions

EVpedia is an integrated database of high-throughput datasets from EVs derived from prokaryotes and eukaryotes. This database is scheduled to be updated every six months. This free web-based database should be a useful resource to elucidate fundamental roles of EVs derived from prokaryotes and eukaryotes.

Furthermore, for high-quality EV datasets, the unified criteria for high-throughput datasets are needed. First, the coherent standards for EV preparation should be defined. Although the detailed procedures vary with different studies, most studies commonly used combinations of filtration, differential centrifugation, and density gradient centrifugation methods to purify EV. In addition, coordinated standards for high-throughput data production are required because there are many systems and programs to produce and analyze high-throughput data, such as mass spectrometry-based proteomics, microarray-based transcriptomics, next-generation sequencing-based transcriptomics, and chromatography-based lipidomics (24). Therefore, in order to collect together the dispersed data of EVs, it is crucial that a clear and detailed guideline for the preparation of EVs and analysis of their high-throughput data is set up.
